# Stockpiling Basic Medical Equipment for Public Health Emergencies and “The-Right-Thing-To-Do.” Charting the Ethical Territory

**DOI:** 10.3389/ijph.2022.1604679

**Published:** 2022-05-30

**Authors:** Andrea Martani

**Affiliations:** Institute for Biomedical Ethics, University of Basel, Basel, Switzerland

**Keywords:** epidemic, public health ethics, public health emergencies, preparedness and response plans, justice

Is profiting from private stockpiles in a territory hit by a catastrophe “the-right-thing-to-do” [1]? This is more than a philosophical question; it is a pressing issue in public health where policymakers and practitioners are concerned about stockpiling basic medical equipment for public health emergencies (PHE). PHE are “extraordinary” events that “constitute a public health risk […] through the international spread of disease and … potentially require a coordinated international response” [2]. For example, at beginning of the COVID-19 PHE, protective equipment like masks [3] were in short supply and a Swiss company took advantage of the emergency by selling masks at allegedly inflated prices to the Swiss and German governments, making enormous profit [4]. When a second COVID-19 wave rushed across India in Spring 2021, some argued that the government mishandled stockpiles and debated whether other countries had the duty to restock India’s reserves [5]. Policy decisions about stockpiling basic medical supplies for PHE have generated moral doubts about “the-right-thing-to-do.” During the COVID-19 PHE, ethicists have often reflected on the need to procure and distribute vaccine stockpiles [6], but few have addressed the more general question of stockpiling medical equipment. This editorial thus charts the ethical territory around stockpiling medical equipment for PHE and suggests criteria that public health experts and policymakers can use to decide on “the-right-thing-to-do.”

The ethics of medical stockpiling for PHE have both a “temporal” and a “spatial” dimension. The ethical questions raised by the temporal dimension depend on whether a PHE was predictable or could not have been anticipated and must be considered both for the period before the PHE and during the PHE. Ethical questions are also shaped by a spatial dimension. The ethics of stockpiling may vary, depending on the regions in which medical equipment is needed. These dimensions will be addressed separately, though they are interconnected.

Temporally, let us start with what is ethically at stake before and after PHEs are evident. Before PHEs emerge, the main ethical question is how to deal with uncertainty. PHEs can differ widely, so deciding which and what kind, where and how much medical equipment should be stockpiled is challenging. Kotalik optimistically argues that we can find ethically acceptable answers by “combining expert and public input” [7], but governments are often reluctant to invest in preventive health measures. Moreover, the moral argument that scarce funds should be invested in tackling existing medical problems is intuitively appealing and widespread.

The literature on the ethics of PHE preparedness [8, 9] is limited, but two recommendations relevant for stockpiling can be derived from that basis. First, governments need plans to counter a “just-in-time” culture that cuts inventory and only orders goods when they are needed. Hospitals often take a just-in-time approach to procuring medical supplies [8], which saves money under normal circumstances but can be disastrous during a PHE when goods may be difficult to procure. Second, governments should plan to respond to potential disruption of global supply-chains, so basic social functioning can be guaranteed during PHE [9]. Without basic social functioning, sectors like first responders, police, sanitation and waste disposal services may break down [6].

On the other hand, different ethical questions emerge during a PHE. How do we avoid depleting stockpiles of protective medical equipment to the extent that healthcare personnel face disproportionate risks when fulfilling their duty to care? And how can we distribute available medical stockpiles in a just fashion across the territory of interest? These are questions situated within the “spatial” dimension, and they raise some of the most complex moral doubts about handling PHE stockpiles since they focus on just allocation of scarce resources. First, policy makers must determine how basic medical stockpiles will be divided within healthcare systems. These choice must be made at the level of public health; they are macro-level decisions about where to send stockpiles rather than decisions made by healthcare personnel about which patients to treat in a clinic where there are shortages. Potentially, policy makers might adopt Daniels’ framework of “accountability for reasonableness” [10] and require that allocation decisions: 1) be publicly discussed; 2) have an evidence-based explanation; 3) are open to revision and appeal; and 4) are regulated.

Finding just criteria for deciding how to manage stockpiles across different nations is much more complicated. There are moral arguments for prioritising fellow citizens [11] based on community ties in our country of residence. In this view, a country’s residents may justifiably retain their stockpiles and wait for a more propitious moment to help residents of other countries. Others, e.g., Hassoun [12], argue that residence or nationality has no ethical relevance and that stockpiles should be distributed across countries in alignment with the same principles by which resources are allocated at a national level. Emanuel et al suggested a middle ground [13] for vaccine stockpiles and their arguments can be generally applied to medical stockpiles. They argue it is ethically acceptable for a government to prioritise its own residents, but only insofar as it is necessary to retain stockpiles to keep mortality at “non-crisis” levels and if the government maintains reasonable public health restrictions (e.g., contact tracing and mask obligations) to control the PHE.

Ethicists cannot offer clear guidelines on exactly “the-right-thing-to-do,” but they can help policy makers and the public “become aware of … moral routines and engage in a process of moral inquiry in which … moral presuppositions are reconsidered” [14]. In PHE, this process of moral inquiry requires us to be aware of the “temporal” and “spatial” dimensions of medical stockpiling so we can tackle the issues of uncertainty (e.g., by revising the “just-in-time” culture) and resource allocation (e.g., by deciding how to share stockpiles across countries). By applying this analytical framework, in both its dimensions, public health decision-makers will be better prepared to determine “the-right-thing-to-do.”
